# Advances in melanoma: epidemiology, diagnosis, and prognosis

**DOI:** 10.3389/fmed.2023.1268479

**Published:** 2023-11-22

**Authors:** Shayan Waseh, Jason B. Lee

**Affiliations:** ^1^Department of Dermatology, Temple University Hospital, Philadelphia, PA, United States; ^2^Department of Dermatology, Thomas Jefferson University, Philadelphia, PA, United States

**Keywords:** melanoma, melanoma epidemiology, melanoma genomics, melanoma diagnosis, melanoma classification

## Abstract

Unraveling the multidimensional complexities of melanoma has required concerted efforts by dedicated community of researchers and clinicians battling against this deadly form of skin cancer. Remarkable advances have been made in the realm of epidemiology, classification, diagnosis, and therapy of melanoma. The treatment of advanced melanomas has entered the golden era as targeted personalized therapies have emerged that have significantly altered the mortality rate. A paradigm shift in the approach to melanoma classification, diagnosis, prognosis, and staging is underway, fueled by discoveries of genetic alterations in melanocytic neoplasms. A morphologic clinicopathologic classification of melanoma is expected to be replaced by a more precise molecular based one. As validated, convenient, and cost-effective molecular-based tests emerge, molecular diagnostics will play a greater role in the clinical and histologic diagnosis of melanoma. Artificial intelligence augmented clinical and histologic diagnosis of melanoma is expected to make the process more streamlined and efficient. A more accurate model of prognosis and staging of melanoma is emerging based on molecular understanding melanoma. This contribution summarizes the recent advances in melanoma epidemiology, classification, diagnosis, and prognosis.

## Introduction

The word melanoma brings about fear among the public, patients, and clinicians alike. Perceived as the deadliest form of skin cancer, the scientific community of researchers and clinicians has made concerted efforts to bring about meaningful changes in morbidity and mortality associated with the cancer. Early on, the focus has been on screening and early detection of melanoma that have resulted in a rapid rise in the incidence of early thin melanomas in the last 50 years in countries of fair skin individuals. The diagnosis and treatment of melanoma have made significant strides in the past decade, which coincided with the understanding of genomic basis of melanomas. The diagnosis of melanoma is no longer solely relied upon histologic interpretation of skin biopsies, but, rather, more precise molecular based ancillary tests have emerged to aid the pathologists. The treatment of advanced melanoma has entered the golden era as targeted personalized therapies have emerged that have significantly altered the mortality rate. In this contribution, the current advances in melanoma epidemiology, diagnosis, and prognosis are reviewed.

## Epidemiology

While the incidence and mortality of most cancers have declined over the past several decades, the incidence of melanoma continues to rise, particularly in the countries of fair-skinned populations of European descent. In 2020, the global burden of melanoma increased to 325,000 cases from 230,000 cases in 2012, a 41% increase (1). The highest incidence of melanoma is observed in Australia and New Zealand (1). In the United States (US), melanoma is the fifth most common cancer diagnosed with an estimated 99,780 new cases and 7,650 deaths in 2022, while 97,920 melanoma *in-situ* new cases are expected, a number that rivals the invasive melanomas (2).

### Incidence and mortality

Invasive melanomas account for about 1% of all skin cancer cases, but they account for over 75% of skin cancer deaths (3). Though melanoma is perceived as a deadly cancer, the overall 5-year survival rate is 93.5% (3). The relative high survival rate of melanoma reflects the high proportion of localized disease (78%) that comprise the newly diagnosed invasive melanomas, which has 5-year survival rate of 99.6% (3). Despite the high survival rate, the small fraction of Stage I disease progression accounts for majority of the melanoma deaths (4). Stage III and IV disease have a survival rate of 73.9 and 35.1% respectively, a significant improvement since the introduction of targeted therapies and immunotherapies (3). In 2015, the 5-year survival rate of Stage IV disease was only 15% in comparison.

According to the Surveillance, Epidemiology, End Results (SEER) Program, the median age of melanoma diagnosis in the US is 66 years old (3). The lifetime risk of developing melanoma in White people is 2.6% (1 in 38), 0.6% (1 in 167) in Hispanic people, and 0.1% (1 in 1000) in Black people (5). The incidence rate is higher in men, a rate that is 1.6 times higher than women. While melanoma can develop at any skin site, the arms and legs are the most common site of involvement in women and the head, neck, back, and trunk are more commonly involved in men (6). African-American patients are more likely to develop melanoma on the plantar feet and other sun protected areas (3). Additionally, African-American patients are likely to have more advanced melanoma at the time of diagnosis and generally have a worse prognosis than their White counterparts.

### Epidemic of melanoma

**Figure 1:**
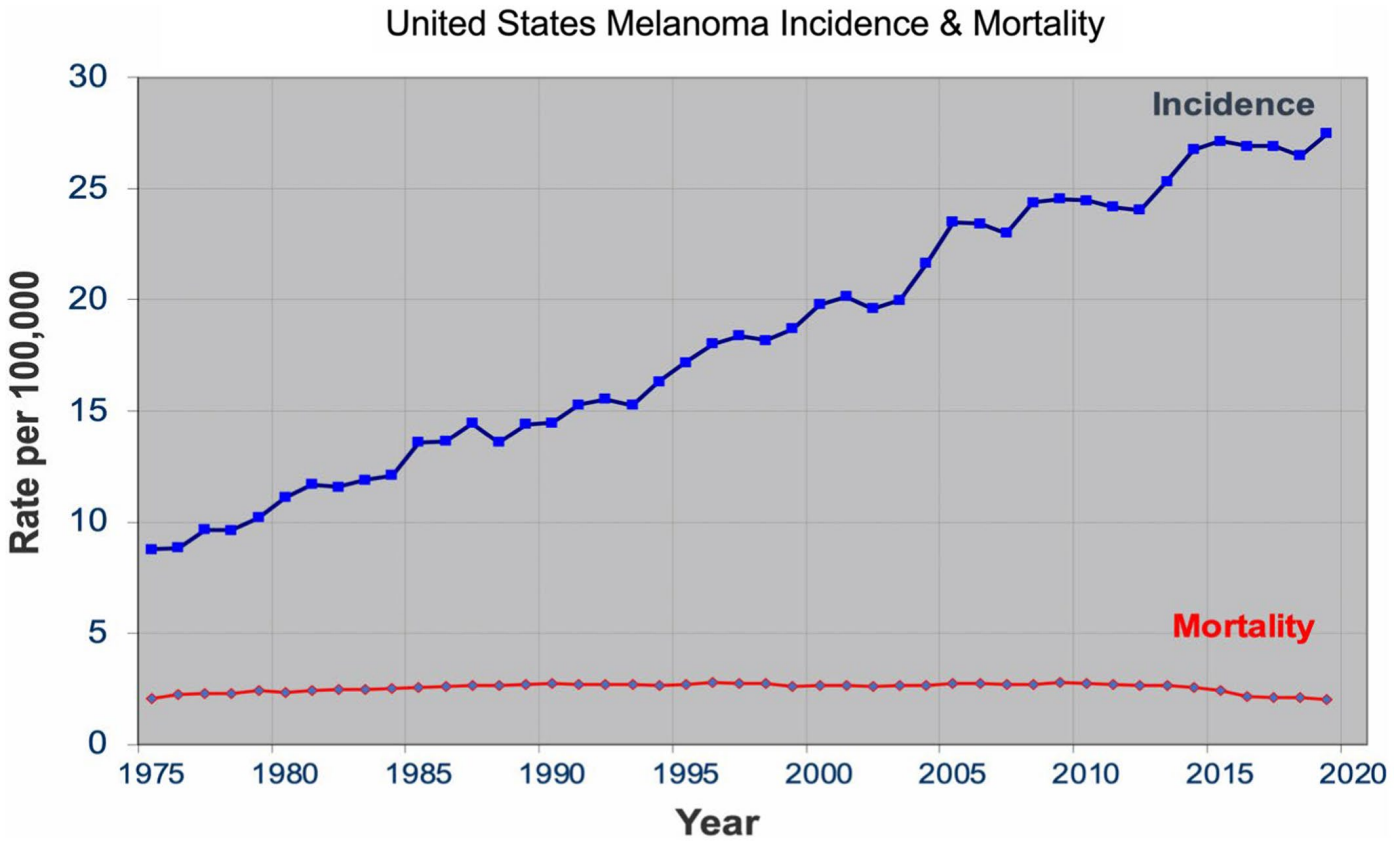
United States melanoma incidence and mortality.

Over the past four decades, there has been a dramatic rise in the incidence of melanoma that has reached epidemic proportions. In the US, a threefold increase in the incidence rate has been observed during this period according to the SEER data all the while the mortality rate remained stable for most of the period (Figure 1) (7). Similar incidence and mortality trends have been observed in developed countries in Europe and Australia over the same period. In the US, the sharp rise in the incidence has coincided with promotion of skin cancer screening and public awareness campaigns in the early 80s. Cancer screening, in general, has an intuitive appeal for clinicians and the lay public: detect cancers early when it’s more curable and manageable to prevent their expected morbidity and mortality. The late A. B. Ackerman urged clinicians and pathologists to diagnose melanomas early at a stage that is small, flat, and curable (8). The widespread adoption of dermatoscopy, a diagnostic technique promoted to detect incipient and incognito melanomas, further contributed to the detection of even earlier stage melanomas (9, 10).

The sharp rise in the incidence of early-stage melanomas without the concomitant rise in mortality has brought the issue of overdiagnosis in the foreground (7, 11–15). Overdiagnosis is defined as identification of a cancer, if left alone, that would not have caused death (16, 17). It is an epidemiologic phenomenon that is easily discernable at the population level, but not at the patient or slide level. The mounting epidemiologic evidence indicates that most of the early melanomas diagnosed represent indolent or biologically benign forms, which are not the obligate precursors to the deadly forms of melanoma, and that the stable mortality reflects the stable incidence of the aggressive melanomas that screening do not capture (7, 18–21). Furthermore, over the past two decades, the incidence of melanoma *in situ* has dramatically increased from 28,600 in 2000 to 101,280 in 2021 in the US (22, 23). Despite the marked increase, no decrease in late disease have been observed. Moreover, they are diagnosed at a later age than the invasive melanomas, further bolstering the argument that they are not obligate precursors of invasive melanomas (24–26).

Effective cancer screening results in a decrease in late-stage disease and mortality. By this metric, cervical and colon cancer screenings qualify as effective cancer screening programs (27). For both cancers, there is a reliable precursor lesion that are screened and removed, human papillomavirus induced cervical intraepithelial neoplasia and colon polyps, respectively. For melanoma, a reliable precursor lesion has been elusive. First described by Wallace Clark in 1978, the dysplastic nevus was promulgated as a precursor lesion to melanoma (28–31), which began an era of close monitoring and their removal that still continues today. The countless biopsies and subsequent excisions of dysplastic nevi, over the past four decades, however, failed to make a difference in the late-stage disease and mortality rate.

The United States Preventive Service Task Force (USPSTF) has consistently given a grade of I for insufficient evidence for or against skin cancer screening, primarily because there are no population-level or randomized controlled trials that demonstrate the benefits of screening (32). There was an initial excitement about the preliminary population data that indicated a decreased melanoma-specific mortality in northern Germany, but the benefit was short-lived and longer follow-up, and the subsequent nationwide population screening had no impact on melanoma-specific mortality (33–35). Accordingly, no major medical societies and organizations in the US have a formal recommendation on skin cancer screening.

According to Welch and coworkers (12), the rapid rise in the incidence is the byproduct of “epidemic of inspection, surveillance, and biopsy of pigmented skin lesions.” The authors recommend curtailing self-referral of skin biopsy specimens, increasing the threshold to biopsy, particularly small, pigmented lesions, increasing the histopathological threshold in the diagnosis of melanomas, and ceasing all population-based skin cancer screenings. The dermatology community is unlikely to follow these recommendations as perceived benefits of screening and early detection are entrenched in the community and overdiagnosis cannot be perceived at the patient or slide level. Without any pivot in the detection strategy, however, epidemiologic evidence of overdiagnosis is expected to become more pronounced. Thyroid cancer has similar issues of overdiagnosis and has a nearly identical incidence and mortality rate pattern. In 2017, USPSTF gave a grade of D for thyroid cancer screening, which resulted in a decrease in the incidence with a mortality rate that remained unchanged in the subsequent years (36, 37). Without formal evidence of benefit, population skin cancer screening is at risk of receiving a grade of D for discourage screening as did thyroid cancer screening. Opportunities awaits dermatology community to perform the necessary studies that show the benefits of screening, particularly in populations that are at high risk of developing melanoma (38).

## Risk factors

### Environmental

Ultraviolet radiation (UVR) from sun exposure has been firmly established as the dominant environmental factor that increases the risk of developing melanoma (39). Intermittent sun exposure, particularly resulting in blistering sunburns, has been hypothesized to increase the risk of melanoma development (40). Meta-analyses have concluded that the relative risk is approximately 2 for sunburn history and 1.3 for tanning bed history (41, 42). In comparison, smoking and lung cancer have a relative risk of 10–20. The relationship between UVR and melanoma risk is a complex one, a relationship that still needs to be further clarified.

**Figure 2:**
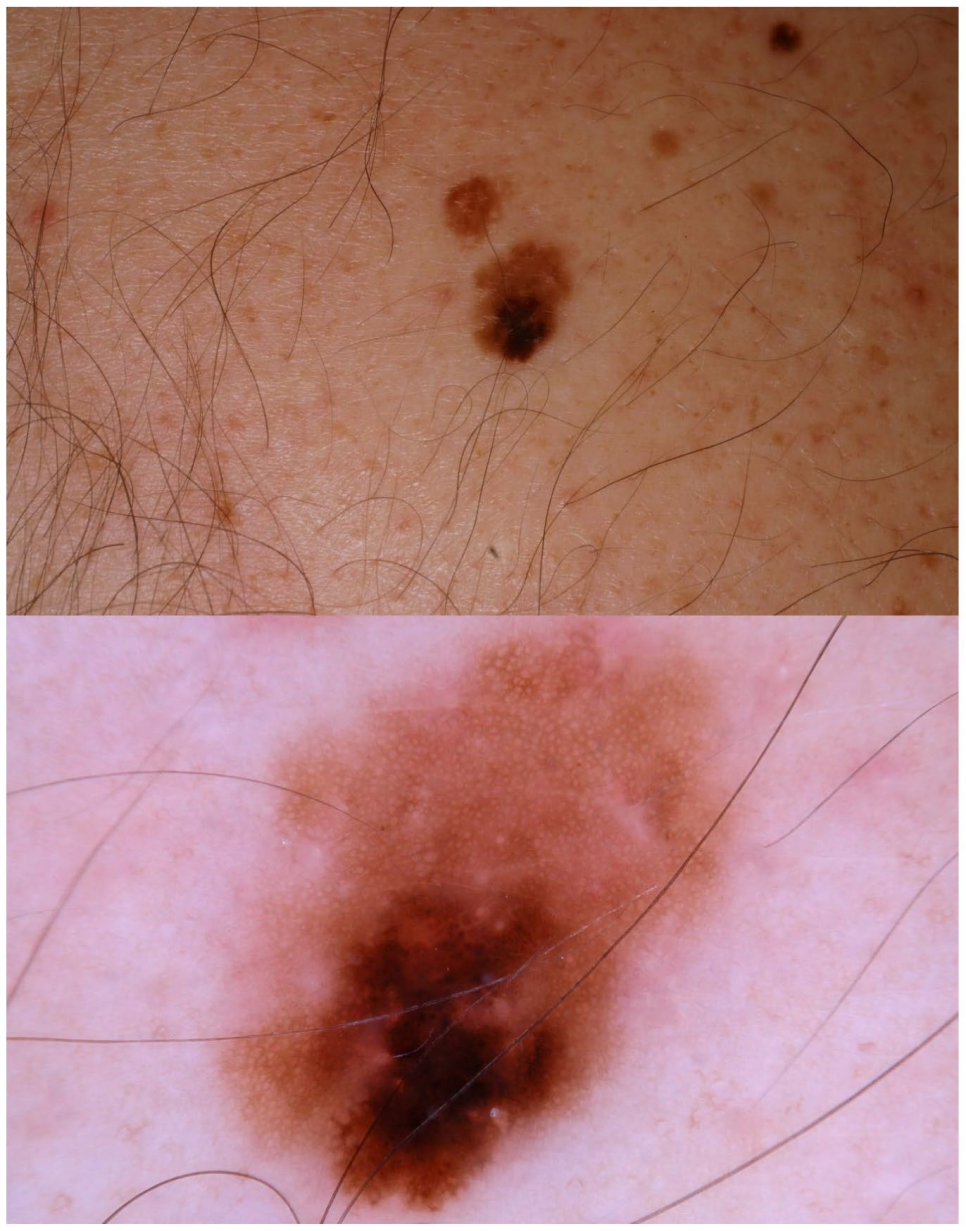
A patient multiple nevi presents with a changing mole. Dermatoscopic image shows an asymmetric melanocytic lesion with regular network and black blotch.

**Figure 3:**
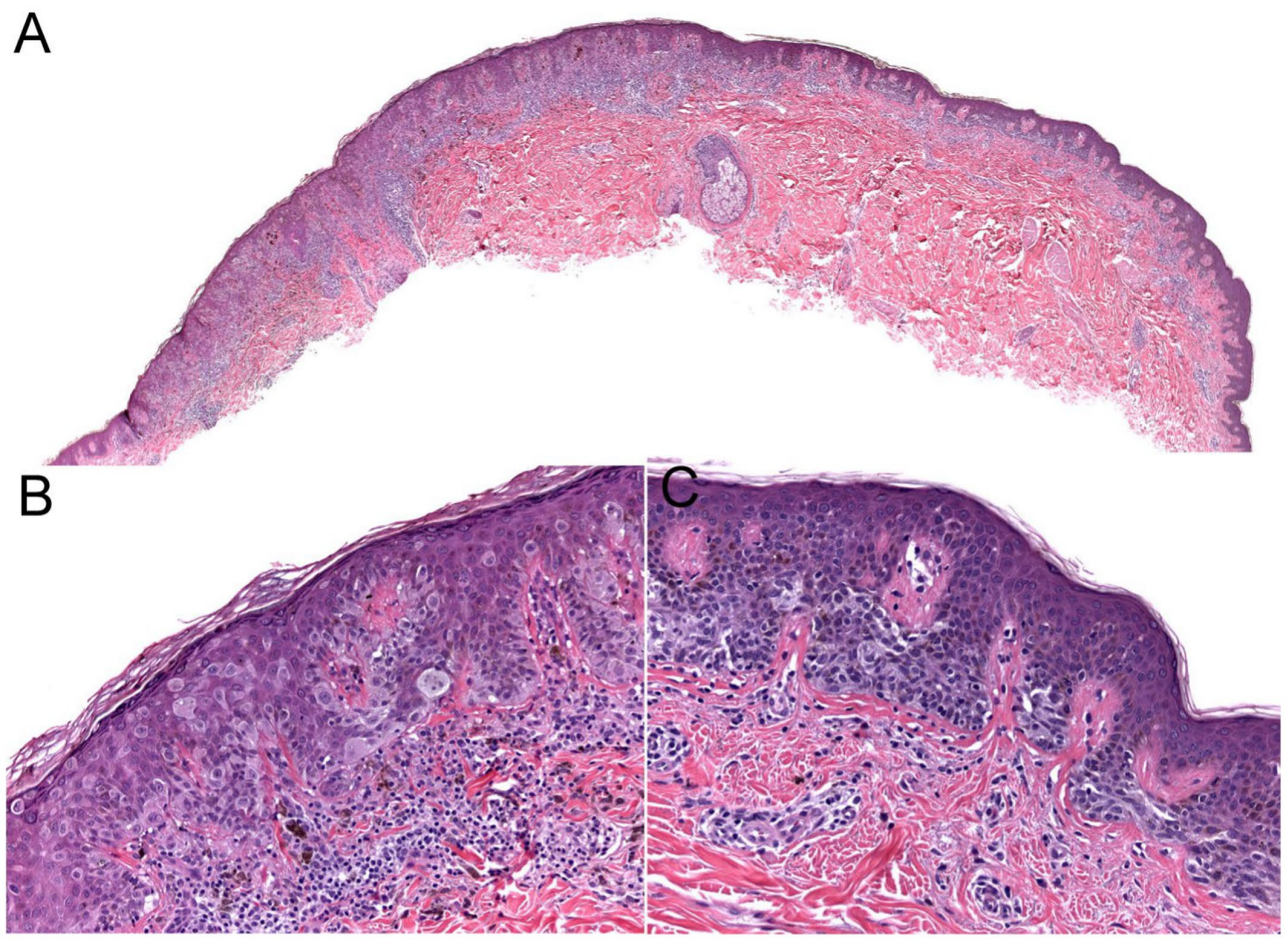
The biopsy of the Figure 2 lesion showing a thin (Stage 1A) superficial spreading melanoma arising in a dysplastic nevus. **(A)** A shave biopsy showing a melanocytic lesion with an asymmetric architecture (20× magnification). **(B)** The left side of the lesion shows the melanoma *in situ* component: large atypical pagetoid melanocytes in pagetoid spread within the epidermis (200× magnification). **(C)** The right side of the lesion shows the nevus component: nested monomorphous melanocytes at the dermoepidermal junction (200× magnification).

**Figure 4:**
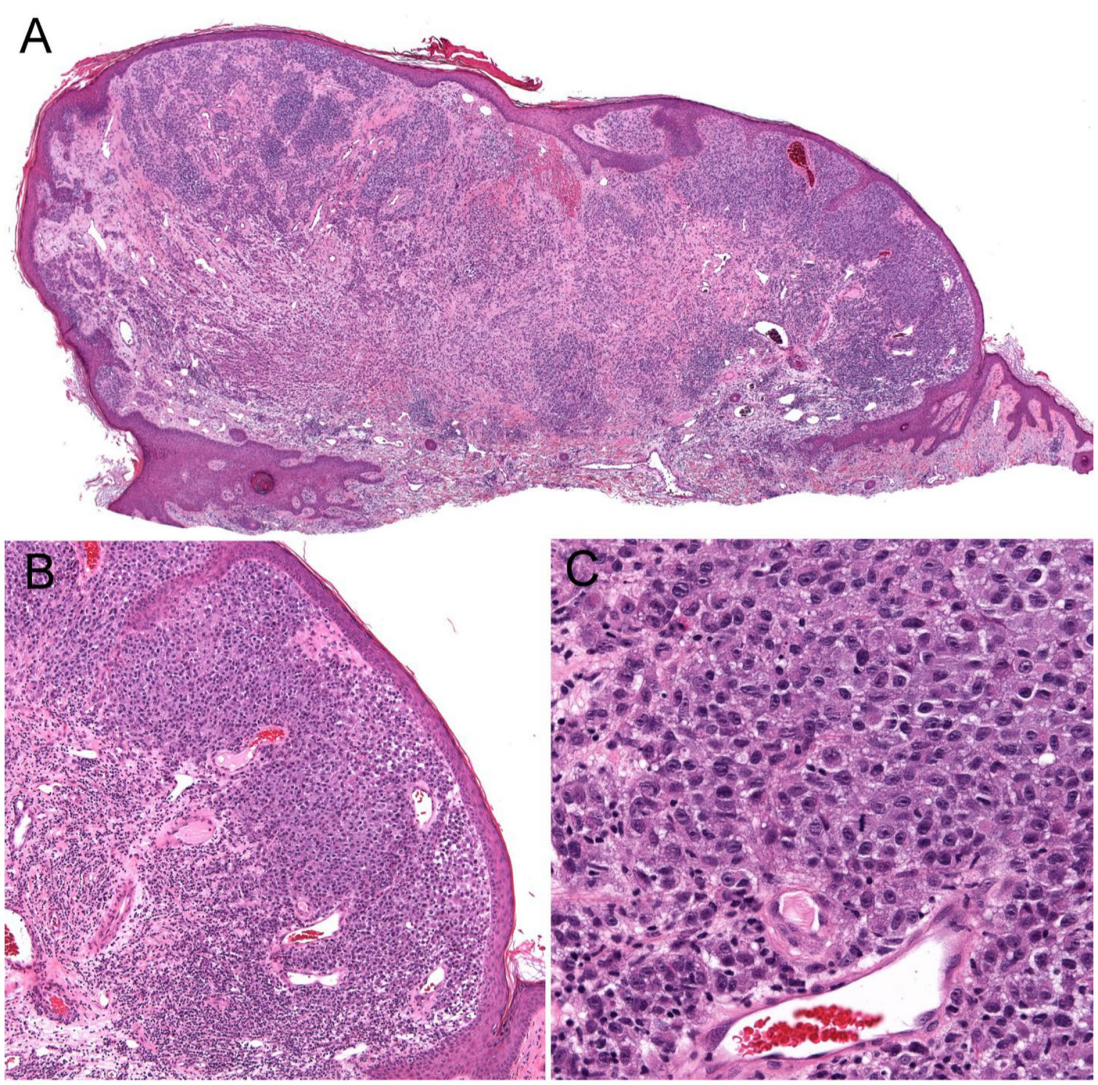
Unsuspected nodular melanoma (Stage 2B) clinically diagnosed as an inflamed skin tag. **(A)** Polypoid asymmetrical melanocytic lesion with irregular distribution of melanocytes (40× magnification). **(B)** Melanocytes arranged in sheets in the superficial dermis (100× magnification). **(C)** Large atypical melanocytes that vary in size and shape with occasional mitotic figures (400× magnification).

Current epidemiologic data suggest the existence of three heterogeneous forms of melanomas: (1) slow-growing melanomas associated with intermittent sun exposure and melanocytic nevi, (2) slow-growing indolent melanomas associated with chronic sun exposure occurring on the head and neck (Figures 2, 3), (3) fast-growing aggressive melanomas minimally associated with sun exposure and melanocytic nevi (Figure 4) (43–45). The fast-growing melanomas are not amenable to screening due to their rapid growth rate. They also elude detection because they do not harbor predictable clinical features, often simulating a benign and malignant non-melanocytic lesions and even inflammatory diseases (46). Nodular melanomas, particularly amelanotic ones, present as rapid growers (46). The current epidemic of melanoma consists of mostly slow growing thin melanomas because of screening efforts, which identify melanomas with early stages that are stretched out much longer in time. Lentigo maligna and superficial spreading type of melanoma fall into this category of melanoma.

### Phenotype risk factors

Phenotype risk factors for developing melanoma includes Caucasian race with fair skin, high nevus count, giant congenital nevus, particularly garment or bathing trunk nevus, and Clark/dyplastic nevus (47, 48). Caucasians have 20 times the risk of developing melanoma compared to Black people (3). Immunosuppression and prior history of melanoma also confer a higher risk.

Giant congenital nevus (>20 cm), particularly garment or bathing trunk nevi, has a significant life-time risk of developing melanoma, ranging from 2.3 to 14% (49). Though some giant congenital nevi may be amenable for prophylactic removal, the garment or bathing trunk nevi are usually too large to remove. Lifetime monitoring for development of melanoma and symptoms due to neurocutaneous melanocytosis is required for these patients.

### Precursor lesion: the dysplastic nevus

In the paradigm of multistep progression of cancer, identification of a reliable precursor lesion is crucial for early detection and reducing the morbidity and mortality associated with the cancer.

In an attempt to follow the successful cervical and colon cancer model and assuming the linear multistep progression paradigm, identification of a precursor lesion for melanoma was sought by clinicians and researchers fighting the battle against melanoma. In 1978, Clark and colleagues described six melanoma prone families where they observed flat melanocytic nevi with irregular border and color variegation in majority of the family members who developed melanoma (28). The authors proposed that these nevi, referred to as B-K moles at the time, have a higher risk of transforming into melanoma. Shortly thereafter, without any formal evidence, these nevi, renamed as dysplastic nevi, received a stamp of approval in a NIH consensus conference of being a marker and precursor to melanoma (30). Over 40 years of practice of close scrutiny and their removal has not resulted in any convincing evidence of their association with melanoma (50, 51). Many authors have concluded that the nevus may serve as a phenotype marker but not a precursor lesion to melanoma (50–54). Some have argued against their precursor status from the outset (55).

### Genetics risk factors

Exciting advances have been made in the discoveries of the genetic underpinnings of cutaneous melanocytic neoplasms, benign and malignant. Germline mutations that significantly increases the life-time risk of developing melanoma include CDKN2A, CDK4, BAP1, TERT, MITF, MC1R, and POT1 (Table 1) (56). These germline mutations underlie the familial or hereditary melanoma *dominant* syndromes, in which melanoma is the predominant cancer of the syndrome. Germline mutations that underlie melanoma *subordinate* or *mixed cancer* syndromes include PTEN, TP53, BRCA1, BRCA2, and XP A-G (57). In these syndromes, other cancers have a higher penetrance rate than melanoma.

**Table 1:** Germline mutations associated with increased melanoma risk.

Gene	Function	Histopathologic subtype	Additional associations
Cyclin dependent kinase inhibitor 2A (CDKN2A)	Tumor suppressor via: (1) p16-mediated inhibition of CDK4 inhibition and phosphorylation of RB (2) p14ARK-mediated inhibition of HDM2 and ubiquitination of p53	Superficial spreading melanoma	Pancreatic, upper GI, and pulmonary cancer Astrocytomas, neurofibromas, schwannomas
Cyclin dependent kinase 4 (CDK4)	Oncogene responsible for downstream inhibition of RB phosphorylation	Superficial spreading melanoma	Pancreatic cancer
Telomerase reverse transcriptase (TERT)	Telomerase component	Nodular melanoma Superficial spreading melanoma	Numerous visceral malignancies
Production of telomeres 1 (POT1)	Component of shelterin complex responsible for telomere regulation	Superficial spreading melanoma	Numerous visceral malignancies
ACD shelterin complex subunit and telomerase recruitment factor (ACD)	Component of shelterin complex responsible for telomere regulation	Superficial spreading melanoma Lentigo maligna melanoma	Numerous visceral malignancies
TERF2 interacting protein (TERF2IP)	Component of shelterin complex responsible for telomere regulation	Superficial spreading melanoma Lentigo maligna melanoma	Numerous visceral malignancies
Melanocyte inducing transcription factor (MITF)	Transcription factor	Amelanotic melanoma Nodular melanoma	Renal cell carcinoma Phenotype of darker hair, fair skin, and non-blue eye color
Melanocortin-1 receptor (MC1R)	G protein coupled receptor for melanocyte-stimulating hormone	Melanoma in specific anatomic sites (e.g., arms)	Phenotype of red hair, freckling, light skin, and UV sensitivity
BRCA1 associated protein 1 (BAP1)	Deubiquinating enzyme and BRCA1 binding partner involved in trascriptional regulation and DNA repair	BAP1-inactivated nevi	Numerous visceral malignancies

Adapted from Toussi et al. (56).

While only 5–12% of melanomas are thought to be hereditary, approximately 40% of hereditary melanomas are attributable to CDKN2A mutations, making CDKN2A the most commonly mutated gene responsible for an autosomal dominant pattern of hereditary melanoma (58). Coding for tumor suppressors p16 and p14 (ARF) which regulate the cell cycle, patients with germline mutation in the CDKN2A gene have a high risk of developing melanoma, glioblastoma, and pancreatic carcinoma (57). Compared to the 2.6% lifetime risk of developing melanoma in the US White population, patients with germline CDKN2A mutation increases that risk to 28 to 76% depending on the presence of other factors. In one study, the risk of melanoma in CDKN2A mutation carriers was approximately 14% by age 50 years, 24% by age 70 years, and 28% by age 80 years (59). In comparison, as one of the mutations for the hereditary pancreatic syndromes, CDKN2A carriers confers a 17% lifetime risk of developing pancreatic cancer (60). Furthermore, while CDKN2A is common somatically mutated in sporadic melanoma, somatic biallelic inactivation of CDKN2A occurs exclusively within invasive melanoma (56). Therefore, the gene continues to represent an important mutational contributor to melanoma development both familial and sporadic.

First discovered in uveal melanoma, mutations in the BAP1 gene interfere with its function as a deubiquitinating enzyme and tumor suppressor (61). Malignancies associated with germline mutations in BAP1 include cutaneous melanoma, ocular melanoma, mesothelioma, renal cell carcinoma, and basal cell carcinoma (61–64). They also may develop small dome-shaped nevi with a spitzoid melanocytes that show loss of BAP1, referred to as BAP1 deficient nevus or Wiesner nevus (65). The vast majority of Wiesner nevus occurs sporadically, and, thus, genetic testing should be based on detailed patient’s history.

TERT, which encodes the telomerase reverse transcriptase subunit of the telomerase enzyme is another important predisposing mutation for the development of melanoma (66). Mutations in the TERT gene allow for the escape of premalignant cells from senesce and apoptosis, contributing to the development of malignancy. First identified in melanoma, TERT mutations have become increasingly identified as one of the most common noncoding mutations in all cancers. Importantly, somatic TERT promoter mutations portend poor prognostic factors, including a higher likelihood of increased tumor thickness and the presence of ulceration, high mitotic rate, and lymph node metastasis (56, 66).

### Gene testing for melanoma

Except for the CDKN2A gene, formal guidelines for genetic testing for mutations responsible for the hereditary melanomas do not exist (57). The “rules of two or three” apply to testing for CDKN2A mutation and not for others (67). Genetic test should be considered for history of three or more primary melanomas and/or pancreatic cancer in geographic areas of high melanoma prevalence and two or more primary melanomas or *in situ* melanomas in areas of low prevalence. History of invasive melanomas in multiple family members at ages earlier than 40 should raise the suspicion of hereditary melanoma syndrome. Leachman and coworkers have outlined more detailed suggestions of screening for germline mutations other than CDKN2A (67). Though technological advances in genomic analysis have enabled discovery of new mutations associated with melanoma and the ease of testing for gene mutations, the actual benefits of the testing and surveillance, in terms of outcomes, are not available.

One of the commonly tested genes is BRCA1 and BRCA2 mutations for evaluation of genetic basis of breast cancers. They both play a role in contributing to the repair of damaged DNA and the destruction of cells with irreparable DNA damage. Although BRCA1 mutations have failed to demonstrate an increased risk of melanoma, BRCA2 mutations have been linked to an increase incidence of melanoma in large breast and ovarian cancer families. An in-depth analysis of published data, however, showed insufficient evidence to warrant increased skin cancer surveillance in these patients without other risk factors (68).

## Melanoma diagnosis

The current clinicopathologic classification of melanoma has been widely adopted and employed in clinical practice for its simplicity and ease of implementation. The classification consists of four major distinct clinicopathologic subtypes with its own corresponding *in situ* lesions: lentigo maligna, superficial spreading, acral lentiginous, and nodular (69, 70). The classification relies heavily on the interpretation of the histopathologic findings, a highly subjective discipline with issues of interobserver reliability (71–74). Though there are melanomas that clearly match the clinical and pathological criteria of a given subtype, many have overlapping histopathologic patterns in the same lesion, making subtyping arbitrary (75, 76). For example, acral lentiginous melanomas have a wide spectrum of histopathologic patterns that encompasses histopathologic patterns observed in the other three subtypes of melanomas. In addition, the classification does not intrinsically incorporate prognostic information. Instead, known extrinsic prognostic factors are added to the pathology report, primarily Breslow depth and ulceration that dictate management. The classification assumes a linear model of progression for all melanomas where melanoma *in situ* lesions are assumed to be the obligate early lesion that becomes invasive and subsequently metastasizes. Though progression of melanomas varies widely, all subtypes of melanomas are treated the same, driven primarily by the thickness of the melanoma.

Recently, the World Health Organization introduced a new classification of melanoma that includes epidemiologic and genomic information in addition to the clinicopathologic criteria. The classification has been expanded to 9 different subtypes that account for the very rare and mucosal melanomas (Table 2) (77). This classification also assumes a linear model of progression in which the melanocytic nevus with the same driver mutation as the melanoma is proposed as the precursor lesion for each subtype. Except for the rare giant or garment congenital nevi where there is a known higher risk of developing melanoma within the nevus, evidence is lacking for the precursor model of progression. The rate at which the precursor nevus acquires the requisite mutations to transform into melanoma is unknown and the rare occurrences of these nevi make it hard to verify their precursor status. The long experience with Clark/dysplastic nevus has not supported the precursor model of melanoma progression (50, 51).

**Table 2:** World Health Organization classification of melanoma.

Relationship with sun exposure	No.	Subtype	Genetic hallmarks
Melanomas arising in sun exposed skin	1	Low-CSD melanoma/superficial spreading melanoma	High frequency of *BRAF* p. V600 mutations
	2	High-CSD melanoma (including lentigo maligna melanoma and high-CSD nodular melanoma)	Predominating mutually exclusive *NF1*, *NRAS*, other *BRAF* (non-p. V600E), and perhaps *KIT* mutations
	3	Desmoplastic melanoma	Recurrent inactivating *NF1* mutations, *NFKBIE* promoter mutations, and several different activating mutations in the MAPK pathway (e.g., *MAP2K1*)
Melanomas arising at sun-shielded sites or without known etiological associations with UV radiation exposure	4	Malignant Spitz tumor (Spitz melanoma)	Mutations in *HRAS* and kinase fusions in *ROS1*, *NTRK1*, *NTRK3*, *ALK*, *BRAF*, *MET*, and *RET*; *CDKN2A* homozygous deletions, *TERT* promoter mutations, and *MAP3K8* fusions / truncating mutations only in aggressive or lethal variants
	5	Acral melanoma (including nodular melanoma in acral skin)	Multiple amplifications of *CCND1*, *KIT*, and *TERT*; mutations of *BRAF*, *NRAS*, and *KIT*; kinase fusions of *ALK* or *RET* in a few cases
	6	Mucosal melanoma	Numerous copy number and structural variations; uncommonly, *KIT* and *NRAS* mutations
	7	Melanoma arising in congenital nevus	In large to giant congenital nevi: *NRAS* mutation; in small to medium-sized congenital nevi, *BRAF* mutations
	8	Melanoma arising in blue nevus	Initiating mutations in the Gaq signaling pathway (*GNAQ*, *GNA11*, *CYSLTR2*, *PLCB4*); monosomy 3 (associated with loss of *BAP1*) and chromosome 89 gains in aggressive cases; additional secondary copy number aberrations in *SF3B1* and *EIF1AX*
	9	Uveal melanoma	Mutually exclusive mutations in the Gaq pathway (*GNAQ*, *GNA11*, *PLCB4*, *CYSLTR2*); *BAP1*, *SF3B1*, and *EIF1AX* mutations during progression

Adapted from Elder et al. (77).

### Melanocytic pathology assessment tool and hierarchy for diagnosis (MPATH-Dx)

While guidelines have been established for melanomas, a standardized management guidelines have not been established for a large group of melanocytic neoplasms for reasons that have plagued the gold standard in diagnosing melanocytic neoplasms—interobserver reliability. In addition, the lack of standardized diagnostic terms for melanocytic neoplasms and disagreements about the fundamental nature of various melanocytic neoplasms have contributed to the confusion among clinicians and patients and the lack of standard management. The local and regional variation on the diagnostic term for the “B-K mole” first described by Clark and colleagues, which includes dysplastic nevus, atypical nevus, nevus with architectural disorder, and Clark nevus, exemplifies the issue of standardization of diagnostic terminology. In 2014, MPATH-Dx schema was introduced to simplify and standardize reporting of melanocytic neoplasms by bring about clarity to classification and management of melanocytic neoplasms, regardless of the different diagnostic terms used (78). The initial version MPATH-Dx consisted of 5 classes with benign and malignant diagnoses with minimal disagreement at the two ends of the classification hierarchy. In early 2023, a new version of MPATH-Dx was published after years of feedback that also accounts for the schema of 2018 WHO classification of melanocytic neoplasms (Table 3) (79). The previous 5 class hierarchy has been simplified into four, essentially removing the original Class II by eliminating the moderately atypical nevus following the WHO classification and moving Spitz nevus into the new Class II group. The new Class I group, referred to as low grade, requires no further treatment while Class II group, referred to as high grade, requires further treatment, which includes a diverse spectrum of melanocytic lesions—high grade dysplastic or atypical nevus, cellular blue nevus, and melanoma *in situ*. As acknowledged by the authors, the MPATH-Dx schema does not escape the issues of inherent subjectivity of pathologic diagnosis of melanocytic neoplasms. While the concordance rates for the two ends of the diagnostic spectrum is good, the concordance rate is poor for thin melanomas, Spitz nevi, and grading of melanocytic lesions (74). In addition, many clinicians will find the margin recommendation of up to 1 cm for melanocytic lesions in Class II vague and arbitrary. Clinicians will look for more precise margin recommendations as 2-, 5-, and 10-millimeter margins make for significant differences in surgery and impact for patients depending on the site and patient’s age. Furthermore, there will be clinicians who will object to the recommendation of removal of routine Spitz, cellular blue, deep penetrating nevi that are included in Class II. Despite these limitations of the scheme, MPATH-Dx is working toward more precise classification, clarity in management, and standardization of reporting that are needed in the diagnostic pathway.

**Table 3:** Melanocytic pathology assessment tool and hierarchy for diagnosis (MPATH-Dx).

Class	Risk of tumor progression	Probability of progression, No. per population	Treatment recommendation	Examples
0	NA	NA	Consider repeat biopsy	Nondiagnostic or unsatisfactory
I: low grade	Very low risk for continued proliferation and progression to invasive melanoma	1 in 10,000 to 1 in 100,000	No further treatment	Common acquired nevi, no atypia Congenital nevi, no atypia Atypical and dysplastic nevi, low-grade atypia Common blue nevi
II: high grade	Low risk for progression to invasive melanoma	1 in 100 to 1 in 1000	Re-excision with margins < 1 cm	Atypical and dysplastic nevi, high grade atypia Spitz nevi, tumors, or melanocytomas, and atypical variants Cellular blue nevi or melanocytomas and atypical variants Plexiform or deep penetrating nevi or melanocytomas Lentigo maligna Melanoma *in situ*
III: melanoma pT1a	Relatively low risk for local and regional metastasis	1 in 10 to 1 in 100	Follow national guidelines (e.g., wide excision with 1 cm margins)	Melanoma AJCC stage pT1a, <0.8 mm Breslow thickness Melanoma pT1a lr (low risk) Melanoma pT1a
IV: melanoma ≥ pT1b	Moderate to increased risk for regional or distant metastasis	1 in 2 to 1 in 10	Follow national guidelines (eg, wide excision with 1–2 cm margins and consideration of sentinel lymph node staging and other therapies)	Melanoma AJCC stage pT1b or greater, ≥0.8 mm Breslow thickness

Adapted from Barnhill et al. (79).

### Clinical diagnosis

The ABCD mnemonic has been a diagnostic aid in the early detection of melanoma since the 1980s, and the recent inclusion of “evolution” as an “E” criterion has been reported to increase its sensitivity. The ABCDE criteria refers to the presence of asymmetry, border irregularity, color variability, a diameter of 6 mm or greater, and evolution or recent change. With the incorporation of ABCDE criteria, diagnostic accuracy of naked-eye examination for melanoma has been estimated to be approximately 65% overall (80–82). The introduction of dermatoscopy has been a major change in the clinical diagnosis of melanocytic neoplasms at the bedside. While the diagnostic technique is not new, the availability of small handheld version of the device coincided with the widespread adoption of dermatoscopy in the modern era. The modern dermatoscope provides a light source, usually a 10× magnification and, more importantly, polarization, which renders the cornified layer translucent, allowing the visualization of subsurface structures (83). Whole new diagnostic criteria of subsurface structures have emerged in diagnosing a variety of inflammatory, infectious, and neoplastic disease of the skin, particularly early melanomas, one of the major objectives of dermatoscopy. Various dermatoscopic criteria have been developed for benign and malignant melanocytic neoplasms. The presence of an atypical pigment network, blue-white veil, atypical vascular patterns, and irregular streaks, pigmentation, globules, or regression structures on dermatoscopy have a higher association with melanoma (84). Diagnostic algorithms that have been developed include the 7-point checklist, Menzies method, and the CASH criteria (84). Each of these algorithms has been shown to increase sensitivity and specificity in melanoma identification. Meta-analyses of large studies have suggest up to 18 and 10% increase in the sensitivity and specificity in the diagnosis of melanoma, respectively (85–87). Major limitations of the studies were that most were retrospective in design evaluated by a group of experts in the field involving images of already managed melanocytic lesions with no impact on management. Accordingly, the more recent Cochrane review (88) concluded that the evidence base of dermatoscopy is limited and “when used by *specialists*, dermoscopy is better at diagnosing melanoma compared to inspection of a suspicious skin lesion using the naked eye alone.” Conclusive evidence was lacking to “explicitly estimate the sensitivity and specificity of dermoscopy, either with or without visual inspection.” Furthermore, despite the enthusiasm and widespread adoption of the diagnostic technique, evidence of desired impact in terms of decreased biopsy rates, cost savings, and improved outcomes of patients are not available. The words of late Carli from 2007 hold true today: “Dermoscopy not yet shown to increase sensitivity of melanoma diagnosis in real practice.” (89) Further studies are needed to validate the widespread use for the diagnosis of melanomas, particularly in the general dermatology and primary care settings.

Recently, a tape-strip test, Pigmented Lesion Assay (DermTech, La Jolla, CA), has been introduced that analyzes the RNA from the stratum corneum for expression levels of RNA Linc00518 (Linc) and PRAME, which are overexpressed in melanomas. The assay also provides the status of TERT, a frequent somatic driver mutation in melanoma. In limited number of studies, the test boasts a greater than 99% negative predictive value, greater than 91% sensitivity, and 70% specificity in the diagnosis of melanoma (90–92). The test has not been widely in use and its role has not been studied extensively. In theory the high negative predictive value should provide reassurance to monitor the pigmented lesion with a negative test. The test also has been promoted to be useful in cosmetically sensitive or difficult to biopsy areas of the skin. Further independent studies and experience are needed to determine whether the promising results are reproducible and the exact role of the test.

### Artificial intelligence

Artificial intelligence (AI) is expected have a profound impact on practice of medicine in the coming years, particularly perceptual specialties such as radiology, pathology, and dermatology as significant advances have been made in image recognition AI algorithms. Jaffe et al. evaluated an AI algorithm that was able to sift through 1,550 images of suspicious and benign skin lesions and identify melanoma with a sensitivity of 100% (93). Importantly, the specificity of the algorithm was found to be 64.8% which was only slightly less than the 69.9% specificity of clinicians. Other studies have shown that artificial intelligence is able to perform similarly to dermatoscopic evaluation in the identification of melanoma (94).

Currently there are no Food and Drug Administration (FDA) approved or cleared AI products in the US for the diagnosis or for the triage purposes of pigmented lesions. The number of commercially available smartphone applications, however, is rapidly growing. Because the diagnostic accuracy of the applications has been inconsistent and unreliable, several reviews have recommended against their use (95–97).

DermAssist, developed by Google, has been CE-marked as a Class 1 Medical Device in Europe with potential for worldwide expansion. The deep learning system within DermAssist was found to be non-inferior compared to 6 dermatologists and superior to 12 primary care physicians and nurse practitioners in providing a diagnosis for a set of 26 common skin conditions (98). When allowed to provide a three-diagnosis differential, the deep learning system was able to achieve a sensitivity of 90% compared to 89% in the dermatologist group and 69 and 72% in the primary care physician and nurse practitioner groups, respectively (98).

As the databases of clinical, dermatoscopic, and histologic images for melanocytic lesions grow, validated training data sets that are more generalizable are expected to emerge, setting the stage for AI augmented practice of dermatology. It is important to note that 90% of the databases used to create the DermAssist software extracted images from patients with lighter skin types; therefore, concerns regarding bias and equal access to the benefits of AI remain to be addressed, particularly in regard to skin of color (99).

### Histopathological diagnosis

Despite the emergence of sophisticated molecular based tests, histopathological diagnosis of melanoma still remains the gold standard. The inherent subjective nature of histologic diagnosis of melanocytic neoplasms has resulted in high rate of discordance among pathologists (71–74). While thick bulky melanomas usually pose no issues, biopsies of ever smaller and thin lesions have further highlighted the problem of interobserver reliability. In the largest iteration of concordance study of melanocytic neoplasms among pathologists, only 25% concordance rate was observed for Spitz and atypical nevi and 45% concordance rate was observed for atypical spitz tumor, severely atypical nevi, and melanoma *in situ*, rates that are unacceptably low to be a valid diagnostic test (74). The low concordance rate among pathologists indicates markedly different thresholds are being applied to a large group of melanocytic neoplasms that have a significant impact on management. With the advances in molecular diagnostics, pathologists are turning to more precise molecular based ancillary diagnostic tests.

Historically, immunohistochemical stains had minimal diagnostic role, having only the confirmatory role of classifying the neoplasm as melanocytic. With insights on molecular signatures of melanomas, several immunohistochemical stains with a more diagnostic role have become available that include PReferentially expressed Antigen in Melanoma (PRAME) and p16. PRAME is overexpressed in melanomas and other cancers. Reflective of its discriminator power, it is included in several gene expression profiling tests for the prognostication of uveal melanoma (Decision Dx-UM), diagnosis of melanoma (myPath Melanoma), and guidance on the decision to biopsy (DermTech). Sensitivity ranging from 67–94% has been reported using PRAME IHC for the diagnosis of melanoma (100–104). For spindle cell desmoplastic melanomas, S100 and SOX10 continues to play a key role in their diagnosis as lower sensitivity ranging from 20 to 35% was observed (104, 105). Other useful IHC stains in the diagnosis of melanoma include p16. The loss of p16 expression, the product of CDKN2A gene, strongly correlates with the diagnosis of melanoma that can be demonstrated with the available IHC stain (106). Differentiating Spitz nevus from spitzoid melanoma, however, is not always helpful (107). Immunohistochemical stains have emerged for detecting the status of BRAF, BAP1, and cKit for the guidance role in targeted therapies.

### Molecular diagnostic tests

Current molecular diagnostic tests for melanoma available to pathologists include comparative genomic hybridization (CGH), fluorescence *in situ* hybridization (FISH), gene expression profiling (GEP). CGH identifies chromosomal copy number variations, including the deletion or multiplications of chromosomal segments (108). The technique involves DNA labeling with fluorochromes that subsequently allow for comparison with reference DNA to highlight genomic areas with gains or losses of DNA material. Advent of single-nucleotide polymorphism arrays allows for targeting genetic loci to the resolution of specific point mutations within the genome of melanocytes, allowing for the identification of loss of heterozygosity, even in chromosomal copy-neutral mutations which are missed with traditional CGH (109). Early CGH studies demonstrated that over 95% of melanomas demonstrated chromosomal number abnormalities in contrast to only 13% of benign nevi (109, 110). More recent studies have demonstrated the utility of CGH in differentiating melanoma from traditionally diagnostically challenging melanocytic entities, such as cellular blue nevi and Spitz nevi. Spitz nevi have been shown to demonstrate only isolated chromosomal number abnormalities at limited loci, while spitzoid melanomas demonstrate multiple copy number abnormalities in various segments (110).

In contrast to CGH that analyzes the whole genome, FISH allows for visualization of gains and losses of specific genomic segments. As normal somatic cells are expected to have two copies of any specific chromosome or chromosomal segment, the presence of more or less than two fluorescent signals indicates the presence of a chromosomal number abnormality. FISH has shown promising results in the differentiation of unequivocal lesions, including conjunctival nevi, epithelioid blue nevi, and, in particular Spitz nevi, and their respective melanoma counterparts with reported sensitivity of 83% and specificity of 94% (111).

While the original FISH assay utilized four probes targeting 6p25 (RREB1), 6q23 (MYB), 11q13 (cyclin D1), and Cep6, two additional probes targeting CDKN2A (9p21) and MYC (8q24) were added, which has increased sensitivity and specificity to 94 and 98%, respectively (112). Although FISH offers a greater ease-of-use and less tissue and labor requirements, CGH has been found to be more sensitive and specific given its ability to assay the entire genome. The high cost and false-positives are additional shortcomings of FISH assays, particularly in lesions that demonstrate polyploidy, such as Spitz nevi which can demonstrate tetraploidy, thus triggering a false positive result (110).

Leveraging real-time reverse transcription-polymerase chain reaction (qRT-PCR) technology, myPath (Castle Biosciences, Friendswood, Texas), a GEP test, has become available for pathologists to aid in the diagnosis of ambiguous melanocytic neoplasms. The assay analyzes the expression of 23 genes that includes PRAME and S100. The assay returns a numerical score that corresponds to likely *benign*, *likely malignant* or likely *indeterminate*. Retrospective validation studies have yielded sensitivity and specificity as high as 94 and 96%, respectively, in unambiguous melanocytic lesions (113–115). A significantly lower sensitivity in the 50% range was observed in studies in which ambiguous melanocytic lesions were evaluated (116, 117). Larger prospective studies on ambiguous melanocytic neoplasms are needed to demonstrate the utility and reliability of the ambiguous lesions for which the test was intended.

### Artificial intelligence

Artificial intelligence augmented practice of dermatopathology is in its nascent stage. Several studies suggesting that performance AI is equal to or better than experienced pathologists have been published in the diagnosis of melanoma in artificial study settings (118–123). To harness the potential of AI in dermatopathology, some barriers need to be solved. Application of AI requires digitalization of slides, cost of which have prevented most laboratories adopting digitalization of the laboratory workflow. Generalizability of results requires large, validated data training sets. Most published studies use proprietary small data training sets that may not be generalizable. Lastly, AI cannot solve the issue of diagnostic discordance issue among dermatopathologists for melanocytic lesions, which will continue to be a barrier in the training and application of AI models (124).

## Prognosis and staging

Clinician have long relied on American Joint Committee (AJCC) on Cancer staging guidelines. The 8th edition of the AJCC melanoma staging system was implemented in 2018. The primary determinant of the localized stage is the Breslow depth of the melanoma and ulceration. Breslow depth is measured from the granular layer of the epidermis down to the greatest depth of the melanoma. One of the biggest changes from the 7th edition is the change in the definition of T1a and T1b. While the cutoff for T1a and T1b stage was ≤1 mm in the 7th edition, it was lowered to <0.8 mm in the 8th edition (125). The result of the change directed more patients to a sentinel biopsy. The full impact of directing more patients for a sentinel biopsy in not known, but the rate of sentinel node positivity appears unchanged in one population-based study (126). A more individualized approach was suggested that accounted for clinicopathologic and molecular features.

Based on qRT-PCR technology that can be performed on paraffin embedded sections, several prognostic gene expression profiling assay tests have become available (127). In the US, 31-gene profiling assay (DecisionDx-Melanoma by Castle Biosciences) has been developed to provide prognostic risk stratification independent of the AJCC staging system. The assay predicts the risk of recurrence or metastasis in stage I, II, and III melanoma. The risk stratification scores consist of 1A (low risk), 1B/2A (intermediate risk) and 2A (high risk) (128). Multiple studies have consistently reported that the assay results independently predict metastatic risk, highlighting the utility of the GEP test (128–131).

While clinicians believe that GEP testing may have clinical benefit for patients with stage II and IIIA disease, the controversy has been for testing patients with stage I disease, a group with a very low risk of recurrence and metastasis (128). As stage I disease make up over 70% of new cases melanoma each year in the US, the test has the potential for high utilization for this stage of the disease, which has raised concerns about the high cost of the test ($7,193 per test) with unknown clinical benefit at this time (132). Meta-analysis by Marchetti and coworkers have concluded the performance of GEP tests for stage I disease was poor and highlighted the potential harm for patients in this group (127, 133). More recently, Kangas-Dick et al. reported that the GEP test did not perform better than traditional clinicopathologic prognostic features in predicting melanoma recurrence risk (134). Proponents and opponents of the GEP test all have criticized the methodologies of the studies that oppose their stance. Consensus statements for and against the test have been published (128, 133, 135). Currently, the American Academy of Dermatology (AAD) and National Comprehensive Cancer Network (NCCN) do not endorse the routine use of GEP. To settle the issue of validity and clinical applicability, authors have recommended prospective randomized clinical trial with predetermined end points free of industry sponsorship bias (128, 132, 133, 136).

## Conclusion

The remarkable advances in understanding the genomic underpinning of melanoma are paving the road for a paradigm shift in the approach to melanoma classification, diagnosis, staging, and therapy. The more precise, objective-based classification and diagnosis of melanoma are expected to replace the clinicopathologic one that is currently widely in use. Molecular diagnostics will play a greater role in the clinical and histologic diagnosis of melanoma as validated, convenient, and cost-effective molecular-based tests are expected to emerge. AI augmented clinical and histopathologic diagnosis of melanoma is expected to make the process more streamlined, precise, and efficient. The next iteration of AJCC staging will better reflect the rapid advances molecular basis of prognostication that is expected to be incorporated. The one issue that needs more immediate attention from the dermatology community is overdiagnosis. Though there is no debate on whether the overdiagnosis of melanoma exists, there is debate as to the degree. Epidemiologic evidence all but indicates a significant degree of overdiagnosis, providing a compelling reason for a shift in strategy from the current approach to melanoma detection. The immediate need is to identify the small fraction of aggressive melanomas within the sea of indolent early melanomas that are being detected today. The resolution of this knowledge gap requires appropriate attention both in terms of funding and research.

## Author contributions

SW: Writing – original draft, Writing – review & editing, Data curation, Formal analysis. JL: Conceptualization, Supervision, Writing – original draft, Writing – review & editing.
